# Difficulties Choosing Control Points in Acupuncture Research. Response: Commentary: Differential Cerebral Response, Measured with Both an EEG and fMRI, to Somatosensory Stimulation of a Single Acupuncture Point vs. Two Non-Acupuncture Points

**DOI:** 10.3389/fnhum.2016.00404

**Published:** 2016-08-11

**Authors:** Till Nierhaus, Daniel Pach, Wenjing Huang, Xiangyu Long, Vitaly Napadow, Stephanie Roll, Fanrong Liang, Burkhard Pleger, Arno Villringer, Claudia M. Witt

**Affiliations:** ^1^Mind-Brain Institute at Berlin School of Mind and Brain, Charité – Universitätsmedizin Berlin and Humboldt-UniversityBerlin, Germany; ^2^Department for Neurology, Max Planck Institute for Human Cognitive and Brain SciencesLeipzig, Germany; ^3^Institute for Social Medicine, Epidemiology, and Health Economics, Charité – Universitätsmedizin BerlinBerlin, Germany; ^4^Acupuncture Moxibustion and Tuina School, Chengdu University of Traditional Chinese MedicineChengdu, China; ^5^Department of Radiology, Athinoula A. Martinos Center for Biomedical Imaging, Massachusetts General Hospital, Harvard Medical SchoolCharlestown, MA, USA; ^6^Department of Radiology, Logan UniversityChesterfield, MO, USA; ^7^Institute for Complementary and Integrative Medicine, University Hospital ZurichZurich, Switzerland

**Keywords:** somatosensory stimulation, functional magnetic resonance imaging (fMRI), electroencephalography (EEG), acupuncture, background rhythm, functional connectivity

The existence of point specificity in acupuncture is still controversial (Choi et al., 2012). Therefore, a thoughtful choice of control points is very important when evaluating point specific effects in acupuncture research.

The standard method of choosing a control for an acupuncture point based on the theory of traditional Chinese medicine is to choose a location on the middle line between two meridians (Yang et al., 2009). However, it is still controversial whether an acupuncture point resembles a specific point (Li et al., 2015) on a meridian or a larger area on the skin surrounding the meridian (meridian skin area; Lin, 1991). Therefore, we think a control point should be located (1) between two meridians and (2) outside the meridian skin area of the acupuncture point under investigation.

However, biomedicine does not well support the existence of meridians (Litscher, 2014). Additional criteria for control point selection can be based on modern anatomy and physiology. This could take into account dermal, muscular, and neural components, as well as connective tissue and chemical aspects. Dermatome and myotome maps, which depict innervation of the skin or muscle by individual spinal cord segments, might help to choose control points that are located in different anatomical areas than the acupuncture point under investigation.

Recently, we designed a blinded study to compare brain activity changes associated with needling at one acupuncture point (ST36) and two control points (Nierhaus et al., 2015). One control point was close, the other distant to the acupuncture point. We selected the control points according to five criteria primarily based on meridian information of traditional Chinese medicine, but also incorporating the perspective of biomedicine by including dermatome map information: (1) control points are not known acupuncture points, (2) control points are not located on a meridian, (3) control points are not located in the meridian skin area of the acupuncture point under investigation (i.e., ST36), (4) the control point close to the acupuncture point under investigation (CP1) is located in the same dermatome as the acupuncture point, (5) the distant control point (CP2) is located in a different dermatome.

Especially the selection of CP1 (close to the acupuncture point ST36) was difficult when following these pre-defined criteria (see Box 1). Based on criteria 4 and 3, we located CP1 in the same dermatome and outside the meridian skin area of ST36. This point is located in a different myotome than ST36, however myotome map information was no criteria for our selection.

Box 1 **Discussion of control point selection for acupuncture point ST36 in a former neuroimaging study by Nierhaus et al. (2015).**

ST36 is located on the Stomach meridian within the Stomach meridian skin area (Lin, 1991) and in dermatome L5 (Drake et al., 2015). The neighboring meridian to the Stomach meridian within dermatome L5 is the Gallbladder meridian. However, a control point for ST36 between the Gallbladder and the Stomach meridian would be within the meridian skin area of ST36. Therefore, we had chosen CP1 on the midline between the Gallbladder meridian and the next meridian—the Bladder meridian. Our choice was based on the dermatome map of Gray's anatomy for students (2nd edition; Drake et al., 2009), which showed our CP1 within L5. This is also confirmed by the 3rd edition from 2015 (Drake et al., 2015) and the 12th edition of Grant's Anatomy from 2009 (Agur and Dalley, 2009). The maps of both textbooks' are based on Foerster's scheme of the dermatomes, which correspond well with clinical findings (Lee et al., 2008; Agur and Dalley, 2009). If the 1972's dermatome map of Grant (1972) were used, the ST36 and CP1 points would be located in dermatomes of L5 and S1, respectively (Wong, 2016). The muscles beneath ST36 are tibialis anterior and extensor digitorum longus (Liang et al., 2006), which are innervated by the deep fibular nerve. The muscle under CP1 is the lateral gastrocnemius, which is innervated by the tibial nerve (Wong, 2016). However, it is not clear whether this difference in innervation might result in different cerebral activation patterns, since there is an overlap of spinal nerve roots for common fibular Nerve (L4-S2) and tibial nerve (L4-S3; Drake et al., 2015). In addition to the nerve innervations of the muscles, ST36 and CP1 share the same peripheral nerve innervation of the common fibular nerve (lateral cutaneous of calf; Drake et al., 2015). Thus, the skin region of ST36 and CP1 is innervated by the same peripheral nerve.

An alternative approach for the selection of control points was recently suggested by Wong (2016). This approach is primarily based on the concept of dermatome and myotome and does not include the meridian concept, thus strictly following concepts of biomedicine. Wong suggested a non-acupuncture point close to ST36 as control point CP1, so that needling of both points (ST36 and CP1) would penetrate the same muscle and the same dermatome.

Unfortunately, both our and Wong's approach are associated with difficulties. A CP1 following our approach might result in needling of different muscles. Thus, the acupuncture point and the respective control point might be more different than desired. In contrast, a CP1 following Wong's approach might result in needling of the same meridian skin area for ST36 and CP1. Thus, both the acupuncture and the control point might be more similar than desired.

Another critical point is the choice of the underlying dermatome map. Due to a lack of consensus on the location and size of individual dermatomes (Lee et al., 2008), specific locations might be attributed to different dermatomes depending on the chosen map. This might lead to different conclusions regarding the selection of a control point. Moreover, there is a high variance between individuals resulting in overlapping dermatomes. More evidence based dermatome maps such as presented by Lee et al. (2008) might be a way to limit these problems. However, these maps need further establishment.

Furthermore, acupuncture and control point can affect different tissues, which in turn might affect brain activity. However, the exact mechanisms involved in the local acupuncture effect [cutaneous, subcutaneous, muscular, chemical (adenosine), connective tissue] are still not clear. We suggest to evaluate needle sensation, e.g., MASS index (Kong et al., 2007), for the different interventions and include the results in the analysis of the data.

In sum, the location of control points in acupuncture research can be based on different alternative body maps (meridian, dermatome, myotome). However, accurate definition of these maps seems to be challenging and may differ between participants. Studies with a high sample size might limit the impact of intersubject variability. A combination of both the traditional concept of acupuncture and modern anatomy seems difficult to achieve when selecting proper control points. Therefore, the underlying concept when choosing control points for acupuncture should be described clearly and more research on acupuncture mechanisms is necessary.

## Author contributions

All authors revised the Response letter to the Comment on our original paper by Dr. Wong.

### Conflict of interest statement

The authors declare that the research was conducted in the absence of any commercial or financial relationships that could be construed as a potential conflict of interest.
